# The Human Early-Life Exposome (HELIX): Project Rationale and Design

**DOI:** 10.1289/ehp.1307204

**Published:** 2014-03-07

**Authors:** Martine Vrijheid, Rémy Slama, Oliver Robinson, Leda Chatzi, Muireann Coen, Peter van den Hazel, Cathrine Thomsen, John Wright, Toby J. Athersuch, Narcis Avellana, Xavier Basagaña, Celine Brochot, Luca Bucchini, Mariona Bustamante, Angel Carracedo, Maribel Casas, Xavier Estivill, Lesley Fairley, Diana van Gent, Juan R. Gonzalez, Berit Granum, Regina Gražulevicˇiene˙, Kristine B. Gutzkow, Jordi Julvez, Hector C. Keun, Manolis Kogevinas, Rosemary R.C. McEachan, Helle Margrete Meltzer, Eduard Sabidó, Per E. Schwarze, Valérie Siroux, Jordi Sunyer, Elizabeth J. Want, Florence Zeman, Mark J. Nieuwenhuijsen

**Affiliations:** 1 Centre for Research in Environmental Epidemiology (CREAL), Barcelona, Spain; 2 Universitat Pompeu Fabra (UPF), Barcelona, Spain; 3 CIBER Epidemiología y Salud Pública (CIBERESP), Barcelona, Spain; 4 Institut National de la Santé et de la Recherche Médicale (INSERM), Institut Albert Bonniot (U823), Grenoble, France; 5 University of Crete (UOC), Heraklion, Crete, Greece; 6 Imperial College London (ICL), London, United Kingdom; 7 Veiligheids- en Gezondheidsregio Gelderland Midden (VGGM), Arnhem, the Netherlands; 8 Norwegian Institute of Public Health (NIPH), Oslo, Norway; 9 Bradford Institute for Health Research, Bradford Teaching Hospitals NHS Foundation Trust (BTHFT), Bradford, United Kingdom; 10 Sensing & Control Systems S.L. (S&C), Barcelona, Spain; 11 Institut National de l’Environnement Industriel et des Risques (INERIS), Unit of Models for Ecotoxicology and Toxicology, Paris, France; 12 Hylobates Consulting S.R.L. (HYLO), Rome, Italy; 13 Centre for Genomic Regulation (CRG), Barcelona, Spain; 14 IMIM Hospital del Mar Research Insititute, Barcelona, Spain; 15 Grupo de Medicina Xenomica, Fundación Pública Galega de Medicina Xeómica (SERGAS), CIBERER-CEGEN, Universidade de Santiago de Compostela, Santiago de Compostela, Spain; 16 Center of Excellence in Genomic Medicine Research, King Abdulaziz University, Jeddah, Kingdom of Saudi Arabia; 17 Vytauto Didziojo Universitetas (VDU), Kaunus, Lithuania

## Abstract

Background: Developmental periods in early life may be particularly vulnerable to impacts of environmental exposures. Human research on this topic has generally focused on single exposure–health effect relationships. The “exposome” concept encompasses the totality of exposures from conception onward, complementing the genome.

Objectives: The Human Early-Life Exposome (HELIX) project is a new collaborative research project that aims to implement novel exposure assessment and biomarker methods to characterize early-life exposure to multiple environmental factors and associate these with omics biomarkers and child health outcomes, thus characterizing the “early-life exposome.” Here we describe the general design of the project.

Methods: In six existing birth cohort studies in Europe, HELIX will estimate prenatal and postnatal exposure to a broad range of chemical and physical exposures. Exposure models will be developed for the full cohorts totaling 32,000 mother–child pairs, and biomarkers will be measured in a subset of 1,200 mother–child pairs. Nested repeat-sampling panel studies (*n* = 150) will collect data on biomarker variability, use smartphones to assess mobility and physical activity, and perform personal exposure monitoring. Omics techniques will determine molecular profiles (metabolome, proteome, transcriptome, epigenome) associated with exposures. Statistical methods for multiple exposures will provide exposure–response estimates for fetal and child growth, obesity, neurodevelopment, and respiratory outcomes. A health impact assessment exercise will evaluate risks and benefits of combined exposures.

Conclusions: HELIX is one of the first attempts to describe the early-life exposome of European populations and unravel its relation to omics markers and health in childhood. As proof of concept, it will form an important first step toward the life-course exposome.

Citation: Vrijheid M, Slama R, Robinson O, Chatzi L, Coen M, van den Hazel P, Thomsen C, Wright J, Athersuch TJ, Avellana N, Basagaña X, Brochot C, Bucchini L, Bustamante M, Carracedo A, Casas M, Estivill X, Fairley L, van Gent D, Gonzalez JR, Granum B, Gražulevičienė R, Gutzkow KB, Julvez J, Keun HC, Kogevinas M, McEachan RR, Meltzer HM, Sabidó E, Schwarze PE, Siroux V, Sunyer J, Want EJ, Zeman F, Nieuwenhuijsen MJ. 2014. The Human Early-Life Exposome (HELIX): project rationale and design. Environ Health Perspect 122:535–544; http://dx.doi.org/10.1289/ehp.1307204

## Introduction

Environmental hazards such as ambient air pollution, environmental tobacco smoke (ETS), noise, pesticides, and radiation may lead to serious, chronic pathologies. The fetus and infant are particularly vulnerable to such potential hazards (Barouki et al. 2012; Gluckman and Hanson 2004; Hines et al. 2010). Environmental exposures—preconceptionally, *in utero*, and during early life—may permanently change the body’s structure, physiology, and metabolism (Gluckman and Hanson 2004). Such changes can promote disease long after the environmental exposure has occurred, including across generations. Environmental exposures during fetal or early life have been associated with adverse fetal growth and with developmental neurotoxic, immunotoxic, and obesogenic effects in children; but for many of these associations, evidence has been classified as limited or inadequate (e.g., Bellinger 2013; Gascon et al. 2013a; La Merrill and Birnbaum 2011; Wigle et al. 2008). Neurodevelopmental disabilities, obesity, and asthma are common and highly complex chronic pathologies, and it is hypothesized that improved understanding of how simultaneous environmental risk factors interact among themselves, with individual characteristics (e.g., genetics), and with epigenetics, can help elucidate their causes (Bousquet et al. 2011; Gallagher et al. 2011; Trasande et al. 2009; Van den Bergh 2011). Up to now, the field of environment and child health has almost uniquely focused on single exposure–health effect relationships; there is no global view of how various types of exposures co-exist and jointly affect health.

*The exposome*. The “exposome” concept was first proposed by Wild (2005) to encompass the totality of human environmental (i.e., nongenetic) exposures from conception onward, complementing the genome; it was developed “to draw attention to the critical need for more complete environmental exposure data in epidemiological studies” (Wild 2012). In this concept, the exposome contains several overlapping domains of nongenetic factors contributing to disease risk, including a general external domain (social, societal, urban environment, climate factors), a specific external domain (specific contaminants, lifestyle factors, tobacco, occupation), and an internal environment (metabolism, gut microflora, inflammation, oxidative stress) (Wild 2012). The exposome calls for improvement of often uncertain exposure data, for integration of data on biological mechanisms, and for a more holistic exposure approach in epidemiological studies. Furthermore, it has been proposed that the exposome may serve an important purpose in characterizing not only the complex mixtures of already identified exposures but also, through its untargeted approach and the use of high-throughput “omics” techniques, relevant exposures that have thus far remained unidentified (Rappaport 2011; Rappaport and Smith 2010). There are large challenges in developing the exposome concept into a workable approach, including the consideration of multiple, longitudinal time periods of interest and of temporal variability, the acknowledgement of exposure uncertainty in an exposome study, the integration of omics data, and the development of powerful statistical techniques to analyze the associations between exposome data and adverse health end points.

The HELIX (Human Early-Life Exposome) project has as its general aim to implement tools and methods (biomarkers, omics-based approaches, remote sensing and GIS-based spatial methods, personal exposure devices, statistical tools for combined exposures, and burden of disease methodologies), to characterize early-life exposure to a wide range of chemical and physical environmental factors and associate these with data on major child health outcomes (growth and obesity, neurodevelopment, respiratory health), thus developing an “early-life exposome” approach. The project takes pregnancy and childhood periods (“early life”) as the starting point for developing the life-course exposome. In this review we describe the general design of HELIX and its main challenges. In this manner, we aim to illustrate how the exposome concept may be implemented in a feasible epidemiological study design.

## Project Concept, Objectives, and Study Populations

**Figure 1:**
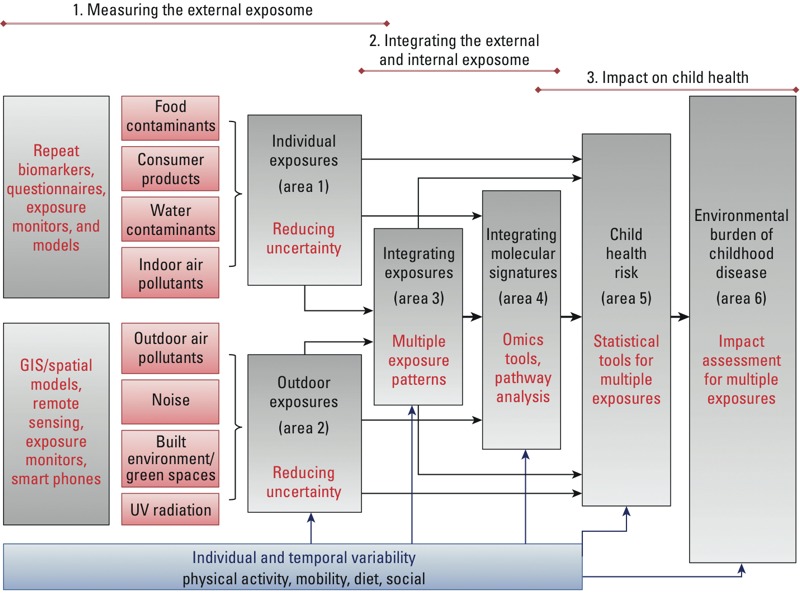
HELIX conceptual framework and interactions between research areas.

**Figure 2:**
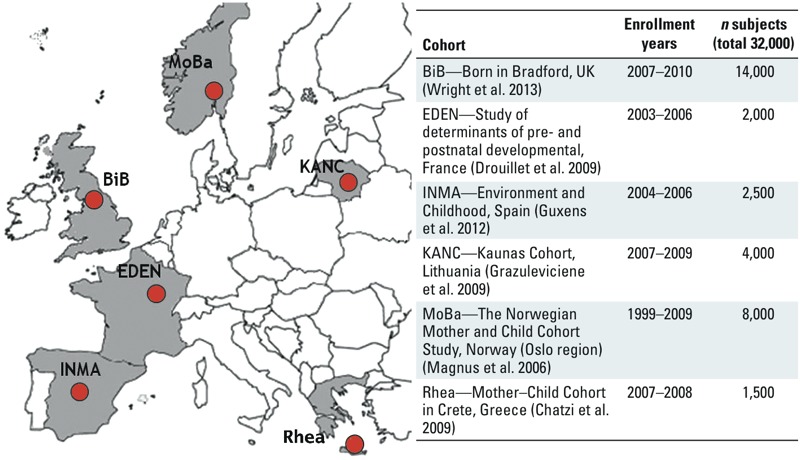
Participating birth cohorts.

HELIX will develop the early-life exposome approach and database in three overlapping steps containing six research areas (Figure 1). A first step will measure the external exposome exposure estimates for a broad range of chemical and physical exposures; a second step will measure the internal exposome (molecular signatures) and integrate the multiple dimensions of the exposome (multiple exposures, multiple time points, individual variability); and a third step will develop the tools and methods to evaluate the exposome’s impact on child health (Figure 1). The project is based in six existing population-based birth cohort studies in Europe (Figure 2). Objectives are the following:

### Step 1: Measuring the external exposome:

To obtain estimates of exposure to persistent and nonpersistent pollutants in food, consumer products, water, and indoor air, during pregnancy and in childhood.
To obtain estimates of chemical and physical exposures in the outdoor environment during pregnancy and in childhood: ambient air pollution, ambient noise, ultraviolet (UV) radiation, temperature, and built environment/green space.

### Step 2: Integrating the external and internal exposome:

To define multiple exposure patterns in the individual and outdoor environment, describe their predictors, and describe uncertainties and variability in the exposures assessed.
To measure molecular signatures associated with environmental exposures through analysis of profiles of metabolites, proteins, transcripts, and DNA methylation in biological samples from the children in the cohorts. Biological pathway analyses will be used to inform analyses of the relationship between multiple exposures and child health.

### Step 3: Impact of the early-life exposome on child health:

To develop a novel multistep statistical approach for the analysis of the association of patterns of multiple and combined exposures and child health outcomes, using agnostic environment-wide association study (EWAS) analysis, structural equation modeling (SEM), and Bayesian profile regression.
To provide exposure–response estimates for the association of multiple and combined exposures with child health, focusing on fetal and childhood growth and obesity, neurodevelopment, and respiratory health.
To estimate the burden of common childhood diseases that may be attributed to multiple environmental exposures in Europe.
To strengthen the knowledge base for European policy in the area of child and environmental health by engaging with, and effectively disseminating HELIX knowledge to stakeholders including those responsible for risk management and mitigation and prevention strategies.

*The birth cohorts*. Six existing longitudinal population-based birth cohort studies in Europe form the basis of the project: BiB (Born in Bradford; United Kingdom) (Wright et al. 2013), EDEN (Étude des Déterminants pré et postnatals du développement et de la santé de l’ENfant; France) (Drouillet et al. 2009), INMA (INfancia y Medio Ambiente; Spain) (Guxens et al. 2012), KANC (Kaunus Cohort; Lithuania) (Grazuleviciene et al. 2009), MoBa (Norwegian Mother and Child Cohort Study; Norway) (Magnus et al. 2006), and Rhea (Greece) (Chatzi et al. 2009) (Figure 2). The cohorts were selected because *a*) they each have a large set of existing longitudinal data from early pregnancy through childhood; *b*) they can implement new follow-up examinations of the children at similar ages (6–9 years), old enough for accurate measurement of the phenotypes of interest for HELIX; and *c*) they can integrate new questionnaires, biosampling, and clinical examinations in their new follow-ups using common protocols. The cohorts have worked together intensively and have pooled data as part of other European Community (EC) projects (Larsen et al. 2013; Vrijheid et al. 2012; see also Supplemental Material, Previous EU projects contributing data and expertise to HELIX, pp. 2–3). The selection of cohorts followed a strategy to obtain data in different regions of Europe.

**Figure 3:**
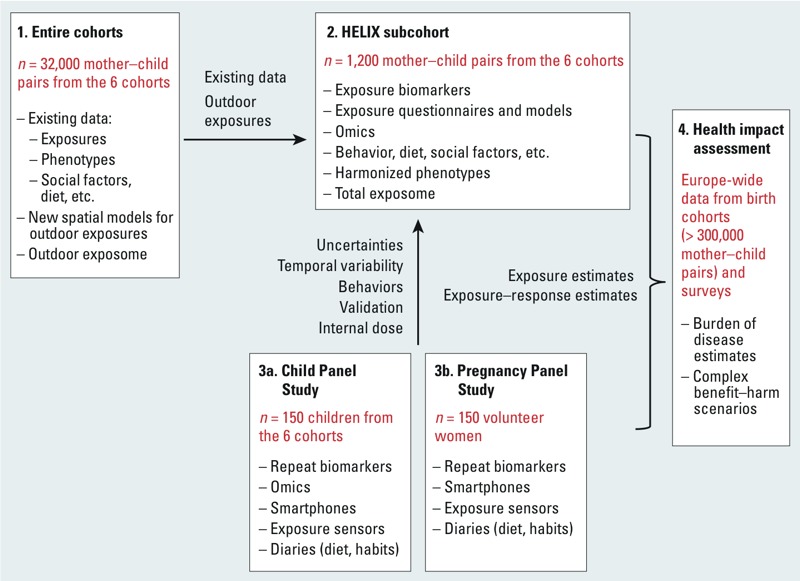
Study design, study populations, and data sources.

*Study populations*. In general, exposure estimates can be obtained in cohort studies for very large numbers of subjects by exposure models and questionnaires, whereas exposure and omics biomarkers can, for cost reasons, be obtained only in smaller numbers of subjects. Assessment of individual exposure variability and validation of exposure models require very intensive data collection that is feasible only in an even smaller number of subjects. For these reasons, HELIX uses a multilevel study design, drawing on nested study populations for four different levels of data collection (Figure 3), as follows:

The entire six cohorts comprising 32,000 mother–child pairs will form the basis of existing data. From this study population we will use existing exposure data such as tobacco use, ESCAPE (European Study of Cohorts for Air Pollution Effects) air pollution land-use regression (LUR) models (Eeftens et al. 2012), water disinfection by-products (DBPs) exposure models from the HiWate project (Health Impacts of long-term exposure to disinfection byproducts in drinking water) (Nieuwenhuijsen et al. 2009), confounder data, and outcome data. Outdoor exposure estimates (research area 2, below) will be applied to these entire cohorts. Risk estimates for the effects of combined outdoor exposures (the “outdoor exposome”) on child health will be obtained in this study population. Harmonization of the existing data will build on protocols and expertise developed in earlier collaborative EC projects (Bousquet et al. 2011; Eeftens et al. 2012; Larsen et al. 2013; Vrijheid et al. 2012; see also Supplemental Material, Previous EU projects contributing data and expertise to HELIX, pp. 2–3). Outcomes that can be harmonized across cohorts include birth outcomes; postnatal growth and body mass index (BMI); self reported wheezing, doctor-diagnosed asthma, and measures of lung function; and neurodevelopment harmonized as five neurodevelopmental constructs (general cognition, language development, motor abilities, socioemotional behavior, and attention deficit/hyperactivity disorder symptomatology) across different age groupings.
1) A HELIX subcohort of 1,200 mother–child pairs (Figure 3) will be fully characterized for the external and internal exposome, including exposure biomarkers during pregnancy and childhood and omics biomarkers during childhood. The impact of the total early-life exposome on child health will be characterized in these 1,200 mother–child pairs. The 1,200 mother–child pairs will be nested within the entire cohorts by selection of 200 pairs from each cohort. Eligibility criteria include *a*) age 6–9 years, 7–8 years, if possible; the age range should be as narrow as possible for comparability of omics analyses and exposure-related behavior; *b*) stored pregnancy blood and urine samples available, and available sample volume sufficient for the analysis of exposure biomarkers detailed in research area 1; *c*) complete address history available from first to last follow-up point; *d*) no serious health problems that, in the opinion of a local clinician, may affect the performance of the clinical testing (e.g., spirometry) or affect the volunteer’s safety (e.g., renal failure, pneumonia). In addition, the selection will consider whether data on important covariates (genetic data, diet, socioeconomic factors) are available. Cohorts with more than the required number of mother–child pairs that meet these criteria will invite subjects at random from the eligible pool. The new follow-up examination will include the collection of new biological samples suitable for all planned biomarker and omics analyses (research areas 1 and 4, below). The collection of two urine samples (one before bedtime and one first morning void) will better capture short-lived biomarker metabolites and provide more stable metabolome coverage than would be achieved with one spot urine sample. Collected blood samples will be processed into appropriate matrices (including whole blood, serum, and plasma) and storage media (for RNA and DNA extraction), and will be rapidly deep frozen under optimized and standardized processing procedures. Variables that can affect omics profiles, such as use of drugs, time of last meal, or physical exercise, will be collected. Trained nurses will carry out health examinations of the children. Examinations include measurements of weight, height, waist circumference, skin folds, blood pressure, and spirometry, and will follow standard operating procedures. Standardized computer-assisted interviews with the mothers will collect information on exposure sources (smoking, cooking, heating, water consumption), physical activity, time activity, diet, social factors, stress, and asthma and allergy. Neurodevelopmental outcomes will be assessed through a battery of internationally standardized, nonlinguistic, and culturally blind computer tests [*n*-back (Vuontela et al. 2003), Attention Network Test (Rueda et al. 2004), Trail Making Test (Lezak 2004), Raven (Raven et al. 1998)]. Parents will complete the Conners Comprehensive Behavior Rating Scales (Conners 1997) and Child Behavior Checklist (CBCL; Achenbach and Rescorla 2000) questionnaires to assess child behavioral problems. Besides the standardization of procedures and questionnaires, the project will implement data collection QA/QC (quality assurance/quality control) through the central training of nurses and field workers—including training workshops with harmonization and reliability exercises (e.g., for the skinfold measurements)—and through visits of coordinators to the local cohorts during the fieldwork to monitor adherence to the standard operating procedures.
2) Panel studies (Figure 3) will collect data on short-term temporal variability in exposure biomarkers and omics biomarkers, on individual behaviors (physical activity, mobility, time activity), and on personal and indoor exposures. A “Child Panel Study” (Figure 3) will include children from the HELIX subcohort (*n* = 150; 25 from each cohort) and will thus be based on the same inclusion criteria. An added requirement for the panel study is that children must be able to wear equipment without destroying it. Invitations will be sent to all families included in the subcohort; but because of the intensive monitoring involved, it is expected that only the most cooperative families will agree to participate, so randomness cannot be guaranteed. Detailed information allowing discussion if the panel study differs from the larger groups will be available. A “Pregnancy Panel Study” (Figure 3) will include 150 pregnant women, 50 from three of the regions under study, and these will be volunteer women from outside the cohorts; mothers from the cohorts cannot be used for this purpose because their pregnancies occurred several years previously. Criteria for inclusion are singleton pregnancy, age ≥ 18 years at the time of start of pregnancy, first visit to be conducted before week 20 of the pregnancy, and residence in the study area covered by the cohort. Study areas will be defined, taking into account the availability of fine-scale air pollution models; as far as possible the areas should correspond to the study areas of the original cohorts, or at least cover similar areas.
3) Subjects in the two panel studies will be followed for 1 week in two seasons. From these subjects we will collect daily urine samples—first morning and last nighttime voids, and from the pregnant women an additional midday void if possible. At the end of each monitoring week, blood samples will be collected following the same procedures as for the subcohort. Subjects or their mothers will complete diaries to collect information on meal times, cosmetics and medication use, and urination frequency for input into the physiologically based pharmacokinetic (PBPK) models (described under research area 1, below). The subjects will carry smartphones and personal monitors, and indoor air and noise monitors will be installed in the homes (research area 2). Additional QA/QC procedures in the panel studies will ensure that the two monitoring periods follow the same procedures in all cohorts and that blood is collected at approximately the same time of the day and under the same conditions in both periods and in all cohorts. This is important to reduce variability in the omics analyses.
4) Health impacts for the larger European population will then be estimated using the exposure levels and dose–response relations from HELIX (Figure 3), together with dose–response and threshold estimates from the literature and prevalence data from European registries and birth cohorts (Vrijheid et al. 2012).

## Measuring the External Exposome

Accurate assessment of environmental exposures (reduction of exposure misclassification) remains an important outstanding challenge for health risk and impact assessment. In developing the exposome concept, this challenge is multiplied because it requires obtaining exposure data for many different exposures. Within the external exposome, a distinction can be made between largely individually assessed exposures such as ETS, water contaminants, persistent organic pollutants (POPs), pesticides, and metals, which are traditionally assessed through questionnaires and/or biomonitoring on an individual basis, and exposures in the outdoor environment such as outdoor air pollutants and noise, where, so far, the residence is taken for estimation of exposure, ignoring mobility.

*Research area 1: individual exposures*. Individually assessed exposures can vary on an hourly or daily to yearly basis. Temporal variability is particularly high for exposures with a short biological half-life and little constancy in the underlying exposure behavior [e.g., bisphenol A (BPA), phthalates, organophosporous pesticides (Bradman et al. 2013; Braun et al. 2011; Philippat et al. 2013; Preau et al. 2010)]. For such exposures, intra- compared with interindividual variability is known to be high, and only many repeat measurements over time may give improved exposure estimates. For more persistent exposures, biomarkers give more long-term exposure estimates that are influenced by changes in diet or behavior, for example, by breastfeeding patterns. Research area 1 will measure exposure biomarkers in the subcohort (*n* = 1,200) in appropriate biological samples newly collected from the children and previously collected from mothers during pregnancy. Biomarkers include POPS—PCBs (polychlorinated biphenyls), dichlorodiphenyldichloroethylene (DDE), hexachlorobenzene (HCB), polybrominated diphenyl ethers (PBDEs), perfluoroalkyl substances (PFASs)—in blood samples, nonpersistent chemicals—phthalates, phenols, organophosphate pesticides—in urine samples, metals in blood, and cotinine as a biomarker of ETS exposure (Table 1). Pre- and postnatal questionnaires will collect information on water consumption habits, which will be combined with information on concentrations of DBPs in drinking water from water companies to obtain estimates of exposure to DBPs. Questionnaires will also collect information on sources of indoor air pollution including ETS, cooking and heating appliances, and ventilation (Table 1). In the panel studies, indoor air pollution will be measured to characterize errors when using exposure information from questionnaires and models. This will be done using passive samplers for nitrogen dioxide (NO_2_) and BTEX (benzene, toluene, ethylene, and xylene), and active PM_2.5_ (particulate matter with diameter ≤ 2.5 μm) cyclone pumps, installed in the home. The panel studies will measure daily repeat biomarkers of the nonpersistent chemicals (phthalates, phenols, organophosphate pesticides) in urine (Table 1); these data will be used to characterize inter- and intraindividual variability in these urine biomarkers, and where possible, correct for the uncertainties in the larger cohort.

**Table 1:** Individual exposures.

Exposure group	Entire cohorts (*n* = 32,000)	HELIX subcohort (*n* = 1,200)	Child Panel Study (1 week in 2 seasons) (*n* = 150)	Pregnancy Panel Study (1 week in 2 seasons) (*n *= 150)
PCB-153, DDE, HCB, PBDE-47	—	Biomarkers: in stored pregnancy blood samples^*a*^ and in newly collected child blood samples.	—	—
PFAS (PFOS, PFOA, PFBS, PFHxS, PFNA)	—	Biomarkers: in stored pregnancy blood samples^*a*^ and in newly collected child blood samples. PBPK models for pregnancy and childhood.	—
Metals (Hg, Pb, and TMS)	—	Biomarkers: in stored pregnancy samples^*a*^ and in newly collected child samples: blood (Pb), urine (TMS), and hair (Hg).	—	—
Phthalates (13 metabolites)	—	Biomarkers: in stored pregnancy urine samples^*b*^ and in newly collected child urine samples (last night and first morning void).	Biomarkers: in daily repeat urine samples. Daily data on diet, cosmetics. PBPK model for DEHP.	Biomarkers: in daily repeat urine samples. Daily data on diet, cosmetics. PBPK model for DEHP.
Phenols (BPA, parabens, TCS, BP3)		Biomarkers: in stored pregnancy urine samples^*b*^ and in newly collected child urine samples (last night and first morning void).	Biomarkers: in daily repeat urine samples. Daily data on diet, cosmetics.	Biomarkers: in daily repeat urine samples over whole week. Daily data on diet, cosmetics.
OP pesticides	—	Biomarkers: in stored pregnancy urine samples^*b*^ and in newly collected child urine samples (last night and first morning void).	Biomarkers: in daily repeat urine samples in two seasons. Daily data on diet and repellent use.	Biomarkers: in daily repeat urine samples in two seasons. Daily data on diet and repellent use.
Water DBPs	Estimates available from previous HiWATE project during and after pregnancy.	New questionnaire in children on water consumption and swimming combined with water company data.	Water consumption diaries.	Water consumption diaries.
Indoor air: BTEX, NO_2_, PM_2.5_	Existing questionnaire data on indoor sources during and after pregnancy.	New questionnaire in children on cooking, heating, cleaning, and ventilation.	Passive BTEX and NO_2_ sampling in the home. Active PM_2.5_ sampling. Questionnaire on cooking, heating, cleaning, and ventilation.	Passive BTEX and NO_2_ sampling in the home. Active PM_2.5_ sampling. Questionnaire on cooking, heating, cleaning, and ventilation.
ETS	Existing questionnaire and cotinine data during and after pregnancy.	New questionnaire in children. Biomarkers: cotinine measurement in newly collected child urine and/or hair samples.	Questionnaire on ETS.	Questionnaire on ETS.
Abbreviations: BP3, benzophenone-3; BPA, bisphenol A; BTEX, benzene, toluene, ethylbenzene, xylene; DBPs, disinfection by-products; DDE, dichlorodiphenyldichloroethylene; DEHP, di(2-ethylhexyl) phthalate; ETS, environmental tobacco smoke; HCB, hexachlorobenzene; Hg, mercury; NO_2_, nitrogen dioxide; OP, organophospate pesticides; Pb, lead; PBDE-47, polybrominated diphenyl ether–47; PCB-153, polychlorinated biphenyl–153; PFAS, perfluoroalkyl substances; PFBS, perfluorobutanesulfonic acid; PFHxS, perfluorohexane sulfonic acid; PFNA, perfluorononanoic acid; PFOA, perfluorooctanoic acid; PFOS, perfluorooctane sulfonic acid; TCS, triclosan; TMS, total metal spectrum. ^***a***^Where measurements are available from previous studies, these will be used. ^***b***^Pooling of ≥ 2 urine samples when available.				

One further source of uncertainty in exposure estimates based on biomarker concentrations is their relationship to the internal biologically effective dose (Sobus et al. 2011). The measured biomarker concentration cannot always be considered as a steady-state concentration (particularly for nonpersistent chemicals), nor as a surrogate for the internal dose of the target tissue and has often not been sampled during the entire critical time window (Bartell et al. 2004; Clewell et al. 2008). Here, modeling the toxicokinetics of the chemical using PBPK may help the interpretation of the measured biomarker data. PBPK models describe the fate of chemicals in the body using individual-specific information about the physiology (age, sex, weight) and the biochemistry (enzyme content) of the individual as well as information on the individual’s behavior (breastfeeding, physical activity, diet) (Beaudouin et al. 2010). In the context of population (epidemiological) studies, PBPK models can be used to simulate exposure during critical time periods in between biomarker measurement points (e.g., Gascon et al. 2013b; Lyons et al. 2008; Ulaszewska et al. 2012). To be relevant, this approach requires detailed input data on individual characteristics and behaviors to minimize assumptions and uncertainties. HELIX will evaluate the use of PBPK modeling to interpret biomarkers of exposure to PFASs [perfluorooctane sulfonate (PFOS), perfluorooctanoic acid (PFOA), and di(2-ethylhexyl) phthalate (DEHP)]. For the PFASs, we propose to relate the biomarker measurement in the child to that in the mother during pregnancy using an exposure scenario that integrates maternal–fetal transfers during pregnancy, transfers via breast milk, and diet during childhood. For DEHP, repeat biomarkers in the panel studies and information on exposure-related behaviors and urination times will be used to evaluate the predictable value of different numbers of biomarker measurements.

*Research area 2: outdoor exposures*. For exposures that are traditionally assessed on the basis of residential location, such as outdoor air pollutants, noise and the built environment, major improvements in exposure assessment and reduction in measurement error can be achieved by collecting information on time–space activity, and, in the case of air pollution, on how much air a person inhales. Knowledge on physical activity, which constitutes a proxy of the inhalation rate (Kawahara et al. 2011), for example, may be integrated with personal air pollution measurements to estimate inhalation dose. New geographic information system (GIS)–based exposure assessments (Beelen et al. 2013; Eeftens et al. 2012), remote sensing (Dadvand et al. 2012), and smartphone technologies (de Nazelle et al. 2013) have made it easier to assess outdoor exposures, and to integrate personal mobility and physical activity data.

Research area 2 will construct a GIS environment for the six cohorts, and will assign exposure estimates for air pollutants, noise, ultraviolet (UV) radiation, temperature, built environment/green spaces. The estimates will build on existing LUR air pollution maps (Beelen et al. 2013; Eeftens et al. 2012), noise maps, UV index and METEOSAT data, green space estimates, as well as walkability, building density, and bike lane map information for the built environment (Table 2). Data from existing regulatory monitors and remote sensing data (e.g., from the Tropospheric Emission Monitoring Internet Service; http://www.temis.nl/) will be used to inform ambient spatial exposure models. The aim is to obtain average exposure estimates for the pregnancy period, and during childhood for different time periods, including 1 day, 1 week, 1 month, and 1 year before the outcome and omics assessment. Smartphones will be worn by the participants in the panel studies to provide geolocation data every second and the metabolic equivalent of tasks (METs) every 10 sec, derived from the built-in accelerometer and GPS (global positioning system) and integrated on the specially developed ExpoApp (Ateknea Solutions Catalonia S.A., Barcelona, Spain). We will then translate these data into activity type (resting, cycling, car travel) and derive inhalation rates. The panel study subjects will also wear electronic wrist band UV dosimeters (Seckmeyer et al. 2011), PM_2.5_ active samplers (DCG4004 sampling pump with GK.05SH Cyclone inlet; BGI Instruments, Waltham, MA, USA), and MicroAthelometers (AE-51; Envirodata, Madrid, Spain) for continuous black carbon monitoring (Table 2). Personal exposure estimates will be used to characterize uncertainties in the spatial exposure models.

**Table 2:** Outdoor exposures.

Exposure group	Entire cohort (*n* = 32,000), for pre- and postnatal exposure periods	Subcohort (*n* = 1,200)	Child Panel Study (1 week in 2 seasons) (*n* = 150)	Pregnancy Panel Study (1 week in 2 seasons) (*n* = 150)
Ambient air pollutants	LUR model for NO_2_, PM_2.5_, PM_10_, PM_coarse_, PM_2.5_ absorbance, PM elemental analyses. Routine monitoring and OMI satellite data for temporal variability.	LUR model for NO_2_, PM_2.5_, PM_10_, PM_coarse_, PM_2.5_ absorbance, PM elemental analyses. Routine monitoring and OMI satellite data for temporal variability.	Inhalation rates and mobility (GPS) data from smartphones. Personal monitoring (24 hr) of PM_2.5_ (and black carbon.	Inhalation rates and mobility (GPS) data from smartphones. Personal monitoring (24 hr) of PM_2.5_ and black carbon.
Noise	Existing municipal noise maps to obtain spatial estimates. Address-based modeling of noise at the most and least exposed facade.	New questionnaires in children on bedroom position, noise perception, etc. Noise estimates based on maps and questions.	Time–activity and mobility (GPS) data from smartphones.	Time–activity and mobility (GPS) data from smartphones.
UV	Remote sensing (satellite) UV radiation maps.	New questionnaires in children on traveling, use of sunscreens, clothes, skin color. UV radiation estimates based on maps and questions.	Time–activity and mobility (GPS) data from smartphones and questionnaires. Personal monitoring using electronic UV dosimeters.	Time–activity and mobility (GPS) data from smartphones and questionnaires. Personal monitoring using electronic UV dosimeters.
Temperature	Remote sensing (satellite) temperature maps (from thermal infrared band) and data from local meteorological stations.	New questionnaires in children on heating and air conditioning. Temperature estimates based on maps and questions.	Time–activity and mobility (GPS) data from smartphones and questionnaires. Personal monitoring of temperature using electronic dosimeters.	Time–activity and mobility (GPS) data from smartphones and questionnaires. Personal monitoring of temperature using electronic dosimeters.
Built environment/green spaces	Normalized Difference Vegetation Index from satellite. Building density, walkability score, accessibility, bike lanes, etc., derived from GIS data.	New questionnaires in children on use of green spaces, public spaces, active transportation.	Time–activity and mobility (GPS) data from smartphones and questionnaires.	Time–activity and mobility (GPS) data from smartphones and questionnaires.
Abbreviations: GIS, geographic information system; GPS, global positioning system; LUR, land use regression; NO_2_, nitrogen dioxide; NO_X_, nitrous oxides; OMI, ozone monitoring ­instrument; PM_2.5_, particles ≤ 2.5 μm in size; PM_2.5_ absorbance, measurement of the blackness of PM_2.5 _filters—a proxy for elemental carbon, which is the dominant light-absorbing substance; PM_coarse_, particles between 2.5 and 10 μm in size; PM_10_, particles ≤ 10 μm in size.				

## Integrating the External and Internal Exposome

*Research area 3: integrating exposures*. Once individual and outdoor exposures have been estimated, research area 3 will use analysis-of-variance techniques, incorporating data from both the HELIX subcohort and the panel studies, to understand the variance components for each key exposure (e.g., arising from diet, physical activity, or time of sampling) and describe the uncertainties in each of the exposure estimates. Statistical techniques such as factor analysis and latent class analysis will be used to create a reduced set of continuous exposure indices based on commonly occurring exposures, while individuals who share similar exposure profiles or “exposomes” will be defined. We will then determine the influence of variables such as diet, socioeconomic status, study region, and seasonality on these exposure indices or profiles. Specific attention will be given to the detection of cohort-specific exposure patterns.

**Figure 4:**
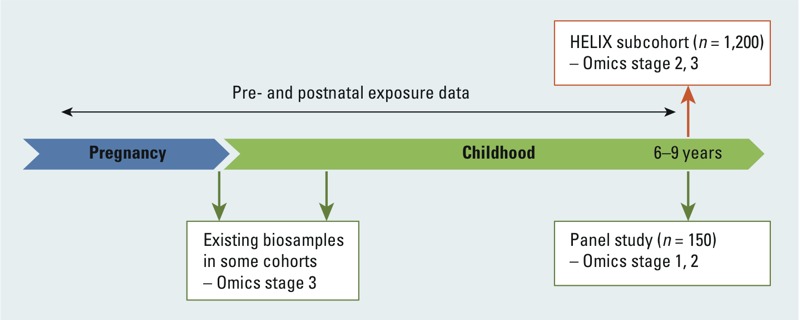
Timeline of the omics analysis.

*Research area 4: integrating molecular exposure signatures*. High-throughput molecular biology “omics” techniques (such as metabolomics, proteomics, transcriptomics, epigenomics) have important potential for broad and untargeted characterization of the internal exposome (Ellis et al. 2012; Hebels et al. 2013). Here, the interest is in the identification of exposure biomarkers and mechanistic pathways. Research area 4 will determine molecular signatures associated with environmental exposures through the measurement of endogenous and xenobiotic metabolite profiles in blood and urine, proteins in plasma, and coding and small noncoding RNAs (including miRNAs; microRNAs) and DNA methylation in whole blood. Omics tools will be employed mainly in the subcohort of 1,200 children with newly collected biosamples at 6–9 years of age; the use of new samples ensures comparability between techniques and cohorts (Figure 4, Table 3; see also Supplemental Material, Detailed description of omics techniques to be used in HELIX, pp. 4–6). The use of a similar time point for all omics techniques also allows integration of the different techniques during data analysis. Genotyping is available already in most of the cohorts and will be completed where needed. Two main limitations in epidemiological studies aiming to use omics biomarkers are tissue and intraindividual variability. Omics profiles are tissue specific, and the tissue of interest can usually not be obtained (e.g., adipose tissue, brain tissue). The focus of HELIX is thus on markers in systemic biological samples (blood, urine) to evaluate the use of omics biomarkers as markers of exposure changes in (larger) epidemiological studies. Omics profiles change over time in the same person; a cross-omics paper with three repeat analyses in 16 subjects over 1 month showed that intraindividual variability for metabolomics and transcriptomics was found to be lower than interindividual variability for almost all the biomarkers (Gruden et al. 2012). However, some sets of markers were highly variable within the same subject and thus cannot be used directly in epidemiological studies. Further, longer time periods of 1 month are likely to give higher intraindividual variability. HELIX will make some progress toward characterizing intra- and interindividual variability in the metabolomics and transcriptomics markers by analyzing repeat biological samples collected in the panel studies in different seasons.

**Table 3:** Omics analyses.^*a*^

Omics technique	Entire cohort (*n* = 32,000)	Subcohort (*n* = 1,200 mother–child pairs)	Child Panel Study (1 week in 2 seasons) (*n* = 150)^*b*^
Metabolomics	—	Untargeted ^1^H NMR spectroscopy and semitargeted UPLC-MS analysis in urine; targeted analysis in serum (using Biocrates Absolute IDQ p180 Kit) in newly collected child samples.	Further analysis of daily urine samples and single serum sample at the end of each week (in winter and summer seasons) to evaluate sources of variation and short-term exposure–omics associations.
Proteomics	—	Targeted analysis in newly collected child plasma samples depending on results of analysis in the Child Panel Study.	Initial iTRAQ and MRM (or similar) analyses in plasma samples collected at end of each week (in winter and summer seasons) to evaluate sources of variation and short-term exposure–omics associations.
Transcriptomics	—	Next-generation sequencing (Ilumina Hiseq2000) or microarray analysis of both mRNAs and miRNAs in newly collected child whole blood samples. In addition, plasma will be collected to analyze miRNAs in the future.	Analysis of blood samples at the end of each week (in winter and summer seasons) to evaluate sources of variation and short-term exposure–omics associations. In addition, plasma will be collected to analyze miRNAs in the future.
DNA methylation	—	Infinium Human Methylation 450 BeadChip for genome-wide methylation analysis of DNA extracted from newly collected child whole blood samples.	Analysis of blood samples at the end of each week (in winter and summer seasons) to evaluate sources of variation and short-term exposure–omics associations.
Abbreviations: ^1^H NMR, proton nuclear magnetic resonance; iTRAQ, isobaric tags for relative and absolute quantitation; MRM, mass spectrometry–based multiple reaction monitoring; miRNA, microRNA; mRNA, messengerRNA; UPLC-MS, ultra performance liquid chromatography–mass spectrometry. ^***a***^Details of the techniques are described in Supplemental Material, Detailed description of omics techniques to be used in HELIX, pp. 4–6. ^***b***^The Pregnancy Panel Study will collect biological samples similar to those of the Child Panel Study. Omics analyses are currently not foreseen in the pregnant women, but samples will be stored for future analysis, e.g., to evaluate whether specific omics findings from the children are replicated in the pregnant women.			

The omics work will be implemented in three general stages:

Stage 1: Study design optimization.Biological samples collected in the panel studies (daily urine samples, two blood samples) will be used to assess detectability of omics markers and the likely sources of variability within and between individuals, using small numbers of subjects. These results will inform the design and interpretation of stages 2 and 3.

Stage 2: Omics–exposure associations in the biological samples newly collected in the subcohort (*n* = 1,200). Primary analyses will evaluate three *a priori* defined exposures (ETS, total POP concentration, and air pollution) and on the specific multiple exposure clusters generated in research area 3. The three exposures have been selected because we have comparatively good long-term exposure estimates, and already there are some data from human omics studies (Bollati and Baccarelli 2010; Hou et al. 2011, 2012; Rusiecki et al. 2008). Power calculations for these analyses are described in the Supplemental Material, Detailed description of omics techniques to be used in HELIX, pp. 4–6, and Table S1. Secondary analyses will examine other exposures. Panel study data will evaluate short-term exposure–omics associations for a range of exposures for which detailed data are collected in the panels: air pollution, noise, UV, and nonpersistent chemicals.

Stage 3. Omics–health associations. Biologically meaningful omics “hits” will be then linked to our main child health end points, similar to the “meet-in-the-middle” approach to biomarker discovery (Chadeau-Hyam et al. 2011). The child health outcomes will be largely continuous outcome scores (BMI *z*-score, cognitive score, lung function). If relevant, reverse causality potential may be evaluated in blood and urine samples available in some of the cohorts at earlier time points (Figure 4).

To analyze, integrate, and interpret the large numbers of data generated by individual omics techniques, HELIX will apply a pathway analysis approach. The biomarkers obtained from the association analysis, in combination with available libraries of biological pathways [Gene Ontology (http://www.geneontology.org/); Kyoto Encyclopedia of Genes and Genomes (http://www.genome.jp/kegg/); Reactome (http://www.reactome.org/); Comparative Toxicogenomics Database (http://ctdbase.org/)] will be used to identify biological pathways affected by the exposures. Identification and representation of biological–toxicological pathways will be done using software such as Ingenuity Pathway Analysis (http://www.ingenuity.com/), Cytoscape (Saito et al. 2012), and Impala (Kamburov et al. 2011). Pathway approaches combining data from different omics techniques (e.g., metabolomics and transcriptomics) are also starting to be developed to search for common pathways determining sensitivity to pharmaceutical and toxic agents (Cavill et al. 2011; Jennen et al. 2011; Kamburov et al. 2011); HELIX aims to use similar approaches.

## Associating the Exposome with Child Health

Finally, one of the greatest challenges of the exposome concept lies in the assessment of its association with health outcomes: How can we integrate multidimensional exposome data to draw meaningful conclusions about (child) health impacts? In general, environmental health studies have considered single exposures or single families of exposure (e.g., atmospheric pollutants, drinking-water pollutants). Notable exceptions of studies that have provided risk estimates for multiple exposures include a cross-sectional EWAS of diabetes (Patel et al. 2010, 2013) and a study by Budtz-Jørgensen et al. (2010) that considered both PCBs and mercury. Statistical analyses that consider many exposure variables simultaneously in a naïve (agnostic) way, such as the EWAS, strongly increase the risk of observing random associations (false positives) because of multiple testing, and of underestimating the global effect of the environment. In developing statistical tools for the analysis of many exposure factors, we should draw important lessons from the achievements but also the limitations (Shi and Weinberg 2011) of the parallel genome-wide association studies (GWAS) field, particularly regarding the probably weak efficiency of purely agnostic approaches, the very large sample sizes required, and the need to use complementary approaches (e.g., pathway analysis) making use of *a priori* information.

Further, the exposome includes evaluation of multiple exposures, omics markers, and outcomes, each with very different temporal scenarios. A challenge in the development of the statistical analysis protocols is to takes these complexities into account. For example, spatial models for the outdoor exposures are constructed for a specific year and can then be extrapolated to relevant time periods (days, weeks, months, or years) backward or forward in time using available monitoring stations data. For persistent pollutants we may assume that biomarkers give estimates over a relatively long time period, whereas for nonpersistent pollutants biomarkers will reflect only very recent exposures; in some cases we may assume a fairly constant exposure pattern depending on the habits underlying the exposure (e.g., cosmetics use, dietary patterns), whereas in other cases exposure variations may be largely seasonal (e.g., sunscreens, pesticides). Neurodevelopment, growth and obesity, and asthma and allergies are each driven by extremely complex multistage developmental processes that take place prenatally and during the first years of postnatal life. Commonly used statistical techniques for high-dimensional data, such as machine learning, dimension reduction, and variable selection techniques, need to be adapted to the longitudinal context by accommodating issues such as time-varying exposure effects, delayed effects, and effects of exposure trajectories over time on outcome trajectories (Buck Louis and Sundaram 2012).

*Research area 5: linking the exposome to child health*. Research area 5 aims to develop a multistep approach that is based on several tools and methods and will produce risk estimates for different types of exposure variables:

Many single-exposure variables: This is an agnostic EWAS approach with no *a priori* information, in which all pairs of exposure–outcome associations will be quantified (using classical regression models), appropriately controlling for false discovery rate (FDR), as is done in GWAS and in the only published EWAS (Patel et al. 2010, 2013); spline and other smoothing models (Slama and Werwatz 2005) will then look for possible thresholds in dose–response relationships.
Combined exposure variables: This is an SEM approach (Budtz-Jørgensen et al. 2010) in which synthetic exposure variables will be built based on previous knowledge summarized by directed acyclic graphs. Several sets of synthetic exposure variables will be considered, including those based on common exposure pathways (e.g., indoor and outdoor air pollutants), on exposure patterns generated by the project, and on knowledge of biological pathways.
Groups of subjects sharing a similar exposome: This approach involves Bayesian profile regression, which aims to identify groups of individuals sharing a similar exposome that at the same time show marked differences according to the health outcome variable of study (Molitor et al. 2010; Papathomas et al. 2011). This is achieved by fitting model-based clustering to the exposure data, while allowing the outcome of interest to influence cluster membership. This technique was used, for example, to identify as a high-risk set for lung cancer a cluster of subjects characterized by their living close to a main road, high exposure to PM_10_ (particulate matter with diameter ≤ 10 μm) and NO_2_, and carrying out manual work (Papathomas et al. 2011). This technique considers the exposome as a whole instead of breaking up risks for individual exposures, and is therefore able to capture effects and complex interactions and combinations of exposures that cannot be detected with the EWAS approach.

SEMs and Bayesian profile regression models will also be used to account for the effects of exposure measurement error and uncertainty, in addition to more classical measurement error models such as regression calibration. The general idea is that these techniques treat exposures not as a fixed value, but as a distribution that can be informed from the repeated measurements (e.g., of nonpersistent pollutants) and personal measurements (e.g., of air pollutants) in the panel studies. As a preliminary step, a simulation study aimed at comparing the efficiency of various study designs and statistical approaches to characterize the impact of the exposome on health will be conducted. With a sample size of 1,200 (subcohort), the agnostic EWAS analysis with control for FDR will have a power of 80% to detect a 3-point difference in a continuous outcome variable with a standard deviation of 15 (as in common neurodevelopment indexes), considering that 15% of the tested exposures will show an association (Liu and Hwang 2007). Higher power will be achieved for exposures available for more subjects (e.g., air pollution) and more hypothesis-driven analyses.

Heterogeneity between cohorts in terms of study design can play a role in the results of these analyses, but it will not be possible to separate these effects from true differences between populations. We will address the issue of cohort differences in two ways: *a*) We will center all exposures using the cohort mean. Analyses conducted with mean-centered variables will remove differences between cohorts and only consider within-cohort differences in exposure. *b*) We will document heterogeneity between cohorts, applying random effects models where applicable, and conduct sensitivity analyses excluding one cohort at the time.

*Research area 6: health impact of multiple exposures*. Finally, research area 6 will estimate the burden of common childhood diseases that may be attributed to multiple environmental exposures in Europe. It will construct scenarios for the health impact assessment, working from traditional one-exposure–one-outcome assessments (e.g., traffic-related air pollution and asthma; mercury and neurodevelopment) to more complex benefit–harm scenarios. For example, given increasing obesity rates, children are encouraged to walk or cycle to school, which may lead to increased energy expenditure and possible reduction in weight and improvement in mental health. However, at the same time, longer duration of exposure to air pollutants, noise, and UV may lead to adverse health effects and higher risks of accidents (Rojas-Rueda et al. 2012). Do the overall benefits outweigh these risks, and what should policy makers do to improve these conditions of active transportation? This work will integrate exposure, uncertainty, and biomarker data obtained in HELIX, risk estimates obtained in HELIX, exposure–response data from the literature, exposure data from other existing birth and child cohorts (Vrijheid et al. 2012) and Europe-wide surveys, and prevalence data from existing health registries/surveys in Europe. Expert workshops will be organized to obtain information.

*Data warehouse*. Data from the previous cohort follow-ups, the new follow-up when the children are 6–9 years of age, the panel studies, and the omics and biomarker analyses will be stored in a common, central database (data warehouse) with common, centrally established QA/QC procedures. Mechanisms to transfer and hold data from across cohorts and other partners will include the identification of agreed data sets, specifications for the data warehouse, data validation, cleaning and harmonization procedures, and the establishment of robust data security mechanisms. Data analysis protocols will be established and performed centrally by a statistical analysis task force. Further, the data warehouse will be established in a format that will allow future uses beyond the project as an accessible resource for researchers external to the project. Procedures for external access will be developed and made public by the end of the project; these will include information regarding data collection, data content, and procedures for data requests.

## Conclusions

There is a strong consensus that new integrative tools and approaches for human exposure and risk characterization are needed to significantly advance environmental risk and health impact assessment and health protection (Cohen Hubal et al. 2010; Lioy and Rappaport 2011; National Research Council 2012). Specifically, new approaches are needed to measure and integrate a wide range of (known and unknown) chemical and physical exposures from different sources and link these to health. The exposome concept may be a useful paradigm for this. HELIX is one of the first attempts to describe the early-life exposome of European populations and unravel its relation to omics markers and health in childhood. As proof of concept, HELIX will be able to evaluate the many challenges in the implementation of the exposome concept and will form an important first step toward the description of the life-course exposome and its health effects

## Supplemental Material

(167 KB) PDF
