# Supplementary material for: The Human Early-Life Exposome (HELIX): Project Rationale and Design
Source: Environ Health Perspect. 2014 Mar 7;122(6):535–44. doi: 10.1289/ehp.1307204 (PMC4048258; doi:10.1289/ehp.1307204)
Supplement: (167 KB) PDF [file ehp.1307204.s001.pdf]

Environmental Health Perspectives doi:10.1289/ehp.1307204

Supplemental Material

The Human Early-Life Exposome (HELIX): Project Rationale and Design

Martine Vrijheid, Rémy Slama, Oliver Robinson, Leda Chatzi,[5] Muireann Coen, Peter van den Hazel, Cathrine Thomsen, John Wright, Toby J. Athersuch, Narcis Avellana, Xavier Basagaña, Celine Brochot, Luca Bucchini, Mariona Bustamante, Angel Carracedo, Maribel Casas, Xavier Estivill, Lesley Fairley, Diana van Gent, Juan R. Gonzalez, Berit Granum, Regina Gražulevičienė, Kristine B. Gutzkow, Jordi Julvez, Hector C. Keun, Manolis Kogevinas, Rosemary R.C. McEachan, Helle Margrete Meltzer, Eduard Sabidó, Per E. Schwarze, Valérie Siroux, Jordi Sunyer, Elizabeth J. Want, Florence Zeman, Mark J. Nieuwenhuijsen

Table of Contents **Page**

Previous EU projects contributing data and expertise to HELIX 2

Detailed description of omics techniques to be used in HELIX 4

Table S1. Sample size required to detect differences in RNAseq experiments. 7

References 8

Previous EU projects contributing data and expertise to HELIX

ESCAPE = European Study of Cohorts for Air Pollution Effects (www.escapeproject.eu/). The ESCAPE project has investigated the long-term effects on human health of exposure to air pollution in Europe. Air pollution (fine particles, particle composition, and nitrogen oxides) exposure assessment was applied at the individual home address level to participants of cohorts from across Europe. ESCAPE provided exposure-response relationships and thresholds for adverse perinatal health outcomes, the development of diseases such as asthma in children; respiratory and cardiovascular disease endpoints in adults; all-cause and cause-specific mortality, and cancer incidence. The project is developing a database for quantitative estimates of the health impacts of long-term exposure to air pollution for all of these health endpoints for the European population (Eeftens et al. 2012, Pedersen et al. 2013).

HiWATE = Health impacts of long-term exposure to disinfection by-products in drinking water (www.hiwate.eu/). The overall aim of this project is to investigate potential human health risks (e.g. cancer, premature births, small for gestational age, semen quality, still birth, congenital anomalies) associated with long-term exposure to low levels of of disinfectants (such as chlorine) and disinfectant by-products (DBPs) occurring in water for human consumption and use in the food industry (Nieuwenhuijsen et al. 2009).

Phenotype = Positive Health Effects of the Natural Outdoor environment in TYpical Populations of different regions in Europe (www.phenotype.eu). Phenotype is investigating the interconnections between exposure to natural outdoor environments (rural and urban) and human health and wellbeing. The underlying mechanisms will investigated for population groups in Lithuania, the Netherlands, Spain and United Kingdom. The project will further examine the effects of different

characteristics of the natural outdoor environment, and address the implications for land-use planning and green space management.

CHICOS = Developing a Child Cohort Research Strategy for Europe (www.chicosproject.eu/). The project aims to improve child health across Europe by developing an integrated strategy for mother-child cohort research in Europe. CHICOS has created an inventory of 77 mother-child cohorts in 21 countries across Europe and has evaluated the state of knowledge on outcomes and determinants from these cohorts, to develop recommendations for research action on child health at a European level (Larsen et al. 2013).

ENRIECO = Environmental Health Risks in European Birth Cohorts (http://www.enrieco.org/) . The project aims to advance knowledge on specific environment and health causal relationships in pregnancy and birth cohorts by providing support to exploitation of the wealth of data generated by past or ongoing studies funded by the EC and national programmes. ENRIECO has extracted environmental exposure response relationships from existing data conducted new work on specific environmental exposure response relationships, and has developed new methods for the standardization of exposure and health outcomes (Gehring et al. 2013; Vrijheid et al. 2012).

MeDALL = Mechanisms of the Development of ALLergy (http://medall-fp7.eu/). This collaborative European project aims to generate novel knowledge on the mechanisms of initiation of allergy from early childhood to young adulthoood, in order to propose early diagnosis, prevention and targets for therapy (Bousquet et al. 2011).

Detailed description of omics techniques to be used in HELIX

Metabolomics

Metabolomics will be employed to screen for both endogenous and xenobiotic metabolites, in both serum and urine samples. The complementary platforms of nuclear magnetic resonance (NMR) spectroscopy and ultra performance liquid chromatography–mass spectrometry (UPLC-MS), using a high resolution quadrupole time of flight (Q-ToF) instrument, will provide broad, untargeted metabolome coverage of urine samples. Subsequently, tandem MS (MS/MS) will be used for structural characterisation and identification of discriminatory metabolites, in concert with NMR and database searching. Furthermore, the Biocrates platform (AbsoluteIDQ p180 Kit), based on analyses using a triple quadrupole mass spectrometer (TQ-S), will be employed in the targeted analyses in serum of approximately 180 pre-selected key endogenous molecules, providing increased sensitivity and absolute quantification for many metabolites.

Proteomics

The examination of the full cellular proteome still remains technologically complex and insufficiently validated to be usable in the context of large-scale population studies. Our proteomics analysis in child blood will therefore adopt a more targeted approach, concentrating on pre-selected proteins (up to 50) suspected to be markers of exposure to contamination, identified either from literature review or chosen to complement and validate 'hits' from other omics analyses such as transcriptomics. We currently plan to initially apply shot-gun proteomics (using iTRAQ technology - http://www.absciex.com) in a reduced number of samples (e.g. 20 samples pooled into each exposure group) in order to find trends in protein abundance between exposure groups and thus identify potential biomarker candidates to be further validated. This will be complemented by mass

spectrometry-based multiple reaction monitoring (MRM) to quantitatively track proteins in a small (20-50) number of individual samples. These experiments will not only evaluate the detectability of such candidate proteins, but most importantly it will assess the variability among donors. We are also currently exploring the use of alternative technologies such as SISCAPA (http://www.siscapa.com/) and MSIA (http://www.thermoscientific.com/en/about-us/promotions/msia-platform.html) that combine the use of antibody-based protein enrichment with mass spectrometric analyses, and the bead-based multiplex platform Luminex (http://www.luminexcorp.com/), in the event that we require greater sensitivity and/or throughput. Based on these studies, candidate proteins will be selected for further analysis in the larger HELIX subcohort.

Epigenomics and transcriptomics

In order to extract RNA from whole blood, including small RNAs, blood will be collected into Tempus tubes (Life Technologies, USA). Total RNA will be quantified using the Thermo nanodrop ND1000 (Thermo Scientific) and quality controlled using the Bioanalyzer Expert 2100 (Agilent). MessengerRNAs (mRNAs) and small RNAs (especially microRNAs (miRNAs)) profiling will be obtained using next generation sequencing platforms (Illumina Hiseq2000). The steps of the sequencing protocol are: 1) library preparation with multiple-indexing (NEBNext Multiplex Small RNA Library Prep Set for Illumina), 2) clustering generation, and 3) sequencing. The coverage needed will be determined at the moment of execution of the project. Other methods, such as microarrays, will be evaluated in case coverage is not sufficient given the actual budget. Technical validation for some transcripts and miRNAs will be performed by qPCR (quantitative polymerase chain reaction). In addition, plasma will be collected to measure miRNAs in future experiments.

DNA from whole blood collected in EDTA tubes will be extracted and quantified. Genome-wide DNA methylation analysis will be selected at the time of execution of the project. Initially we plan to analyze DNA methylation using the Infinium HumanMethylation450 BeadChip Kit from Illumina which evaluates more than 450.000 CpG sites widespread the genome. Although the array covers only 1% of the genome, over 90% of the RefSeq genes are represented. DNA methylation is measured using quantitative "genotyping" of bisulfite-converted genomic DNA. Technical validation for some CpG sites will be performed by pyrosequencing or similar high sensitive techniques.

Main cell proportions in whole blood smears will be counted and taken into account in the statistical analysis, both for epigenomics and transcriptomics.

Power calculations

Power calculations verify that a sample size of 1200 is powered to detect moderate effects (log2-fold change of 1.2 to 1.3) in the case of analyzing 20,000 genes by RNAseq: sample sizes of 200 to 500 per exposure group would be required (standard deviation 0.5-0.6) (Table S1). As we are analyzing different types of data (e.g. count data for RNA-seq, count data for miRNA, continuous data for DNA methylation), we based our computations using existing methods assuming that assumes that genomic data are continuous. The estimates are conservative compared to most of our other omics analyses (e.g. estimated metabolomics 500 metabolites, 30 proteins, miRNA 1,000 genes), apart from methylation where we will analyse 450k CpG sites and thus have lower power. It should be noted that these estimates are based on purely agnostic methods and that pathways analyses will be used to introduce prior knowledge and increase power.

Table S1. Sample size required to detect differences in RNAseq experiments. The setting considers that 20,000 genes are analyzed and a false discovery rate is equal to 5%.

Power	log2-fold change	Standard deviation (σ_d)*	Sample size (N in each exposure group)
80%	1.3	.6	277
80%	1.3	0.5	191
80%	1.2	0.6	501
80%	1.2	0.5	340

From similar previous studies (Lee and Whitmore 2002), it is anticipated that σ_d is around 0.57 ($\sigma = 0.4$).

References

Bousquet J, Anto J, Auffray C, Akdis M, Cambon-Thomsen A, Keil T, et al. 2011. MeDALL (Mechanisms of the Development of ALLergy): an integrated approach from phenotypes to systems medicine. Allergy 66(5):596-604.

Eeftens M, Tsai M-Y, Ampe C, Anwander B, Beelen R, Bellander T, et al. 2012. Spatial variation of PM2.5, PM10, PM2.5 absorbance and PMcoarse concentrations between and within 20 European study areas and the relationship with NO2 - Results of the ESCAPE project. Atm Environ 62(0):303-317.

Gehring U, Casas M, Brunekreef B, Bergstrom A, Bonde JP, Botton J, et al. 2013. Environmental exposure assessment in European birth cohorts: results from the ENRIECO project. Environ Health 12:8.

Larsen PS, Kamper-Jørgensen M, Adamson A, Barros H, Bonde JP, Brescianini S et al. 2013. Pregnancy and Birth Cohort Resources in Europe: a Large Opportunity for Aetiological Child Health Research. Paed Perinat Epidemiol, 27: 393–414.

Lee M-L T and Whitmore GA. 2002. Power and sample size for DNA microarray studies. Stat Med, 21:3543–3570.

Nieuwenhuijsen MJ, Smith R, Golfinopoulos S, Best N, Bennett J, Aggazzotti G, et al. 2009. Health impacts of long-term exposure to disinfection by-products in drinking water in Europe: HIWATE. J Water Health 07(2).

Pedersen M, Giorgis-Allemand SE, Bernard C, Aguilera I, Andersen AN, Ferran Ballester F, et al. (2013) Ambient air pollution and low birthweight: a European cohort study (ESCAPE). Lancet Resp Med, Early Online Publication, 15 October DOI:10.1016/S2213-2600(13)70192-9.

Vrijheid M, Casas M, Bergstrom A, Carmichael A, Cordier S, Eggesbo M, et al. 2012. European birth cohorts for environmental health research. Environ Health Perspect 120(1):29-37.
